# Kaposiform haemangioendothelioma: magnetic resonance imaging features in 64 cases

**DOI:** 10.1186/s12887-021-02573-8

**Published:** 2021-03-03

**Authors:** Suhua Peng, Chunchao Xia, Kaiying Yang, Siyuan Chen, Yi Ji

**Affiliations:** 1 Division of Oncology, Department of Pediatric Surgery, West China Hospital of Sichuan University, #37 Guo-Xue-Xiang, 610041 Chengdu, China; 2 Department of Radiology, West China Hospital of Sichuan University, 610041 Chengdu, China; 3 Pediatric Intensive Care Unit, Department of Critical Care Medicine, West China Hospital of Sichuan University, #37 Guo-Xue-Xiang, 610041 Chengdu, China

**Keywords:** Kaposiform haemangioendothelioma, Kasabach-merritt phenomenon, Magnetic resonance imaging, Radiography

## Abstract

**Background:**

Kaposiform haemangioendothelioma (KHE) is a rare, locally aggressive disorder. The presenting and imaging features of KHE can overlap with other vascular anomalies and tumours. We aimed to analyse the imaging findings of KHE disorder and highlight features most suggestive of this diagnosis.

**Methods:**

The clinical features and imaging findings were retrospectively reviewed in 64 patients with pathological diagnosis of KHE.

**Results:**

Of the 64 patients diagnosed with KHE, 36 patients were < 6 months and 28 patients were ≥ 6 months. The most common presenting features were Kasabach-Merritt phenomenon (KMP, 42.2 %), visible cutaneous lesions (90.6 %), oedema or swelling (43.8 %) and destructive changes or remodelling of adjacent bone (42.2 %). Compared with patients in the group ≥ 6 months, patients in the group < 6 months have higher odds of KMP (*P* = 0.000), infiltrative lesion with ill-defined borders (*P* = 0.044). The group ≥ 6 months have higher odds of destructive changes or remodelling of adjacent bone (*P* = 0.002). In all patients, the lesions in all of the 64 patients were hypointense or isointense compared with muscle on T1-weighted sequences, and hyperintense on T2-weighted or inversion-recovery sequences, nine patients (14.1 %) showed vascularity. There were 28 patients (43.8 %) with characteristic enhancing and infiltrative soft-tissue thickening.

**Conclusions:**

Presence of visible cutaneous lesions with ill-defined borders, destructive changes or remodelling of adjacent bone, severe thrombocytopenia and consumptive coagulopathy should favour the diagnosis of KHE.

## Background

 Kaposiform haemangioendothelioma (KHE) is an aggressive, rare, locally invasive vascular tumour. Most patients present in the first 6 months of life with a characteristic purpuric, cutaneous lesion. Other sites of involvement include retroperitoneum, abdomen, mediastinum and muscle-bone-joint [1, 2]. Pathologically, KHE is comprised of spindled endothelial cells, and immunopositivity for both vascular and lymphatic endothelial markers, immune-negativity for glucose transporter-1 and human herpesvirus-8 [3]. Complications of KHE include Kasabach-Merritt phenomenon (KMP) resulting from platelet activation, trapping and consumption [1]. Clinical and laboratory findings of KMP are specific, whereas imaging also plays an important role for early diagnosis and assessment, especially for KHE without KMP [4].

For evaluation of superficial soft tissue masses in children, ultrasound (US) is often used as the initial diagnostic imaging modality [5, 6]. The consideration of vascular tumours could be suggested by using US. However, a more specific diagnosis might not be made with US because US usually failed to clearly demonstrate the infiltrative portions of KHE. In this regard, magnetic resonance imaging (MRI) is the imaging modality of choice [4]. Previously, different imaging features of KHE have been described. However, detailed analysis was lacking [7, 8]. In this study, we retrospectively analysed the radiological features of KHE, with the aim to improve our understanding of KHE and prevent morbidity and mortality.

## Methods

 This study was approved by the Institutional Review Board of the West China Hospital of Sichuan University. Written informed consent was obtained from the parents of all patients. We conducted a retrospective analysis of all patients with KHE diagnosed from January 2014 and March 2019. The requirement for informed consent was waived due to the retrospective nature of this study. All cases of KHE included in the present study were collected by searching the clinical database at West China Hospital of Sichuan University. A total of 71 patients were diagnosed with KHE according to clinical, pathological, and radiological findings. Physical examination, laboratory investigations and radiological findings were entirely reviewed in all patients. All patients underwent ultrasound examinations before CT or MRI. Seven of these patients were excluded due to lack of radiologic and/or clinical information. Based on the depth of tissue or organ involvement, lesions were classified into three groups: superficial, mixed and deep. Superficial KHEs were lesions involving the dermis, subcutaneous tissue and deep fascia. Mixed KHEs were defined as cutaneous lesions with deep infiltration into muscle, bone, intrathoracic sites or retroperitoneal sites. Deep KHEs were defined as non-cutaneous lesions located in the mediastinum, retroperitoneum, internal organs, and muscle-bone-joint areas [2, 9].

The images were analysed by two radiologists in consensus. They were informed of the histological diagnosis but not the imaging findings. Lesion size and distribution were recorded for individual patients. The maximum diameter of the tumour, adjacent bone changes and anatomical regions were measured. Images depicting masses that passed through more than 2 anatomical regions were considered to reflect multiple anatomical regions involvement. According to the solidity of the mass, cases of KHE were classified into two morphological types: 1, solid mass with or without surrounding infiltrative portions; 2, infiltrative lesion without defined areas of solidity. Furthermore, we retrospectively evaluated oedema/swelling, adjacent fat stranding, signal void (MRI), vascularity, calcification, and haemorrhage. The presence of oedema/swelling was defined as areas showing hyperintensity on T2-weighted images with or without fat suppression and hypointensity on T1-weighted images in comparison to normal muscle, and the subcutaneous fat and deep soft tissue was obviously thickened with reticular stranding [7].

We report the basic descriptive statistics for clinical and imaging characteristics. The differences in the proportions of clinical and imaging characteristic whose lesions discovered were < 6 months versus the differences in the proportions of those ≥ 6 months group were evaluated. Pearson chi-squared test and Fisher’s exact test were used to analyse of categorical variables. Student’s *t*-test was used to analyse continuous variables where appropriate. Statistical analyses were conducted using SPSS 22.0 for Windows (SPSS Inc., Chicago, IL, USA). *P* values less than 0.05 were considered statistically significant).

## Results

**Fig. 1:**
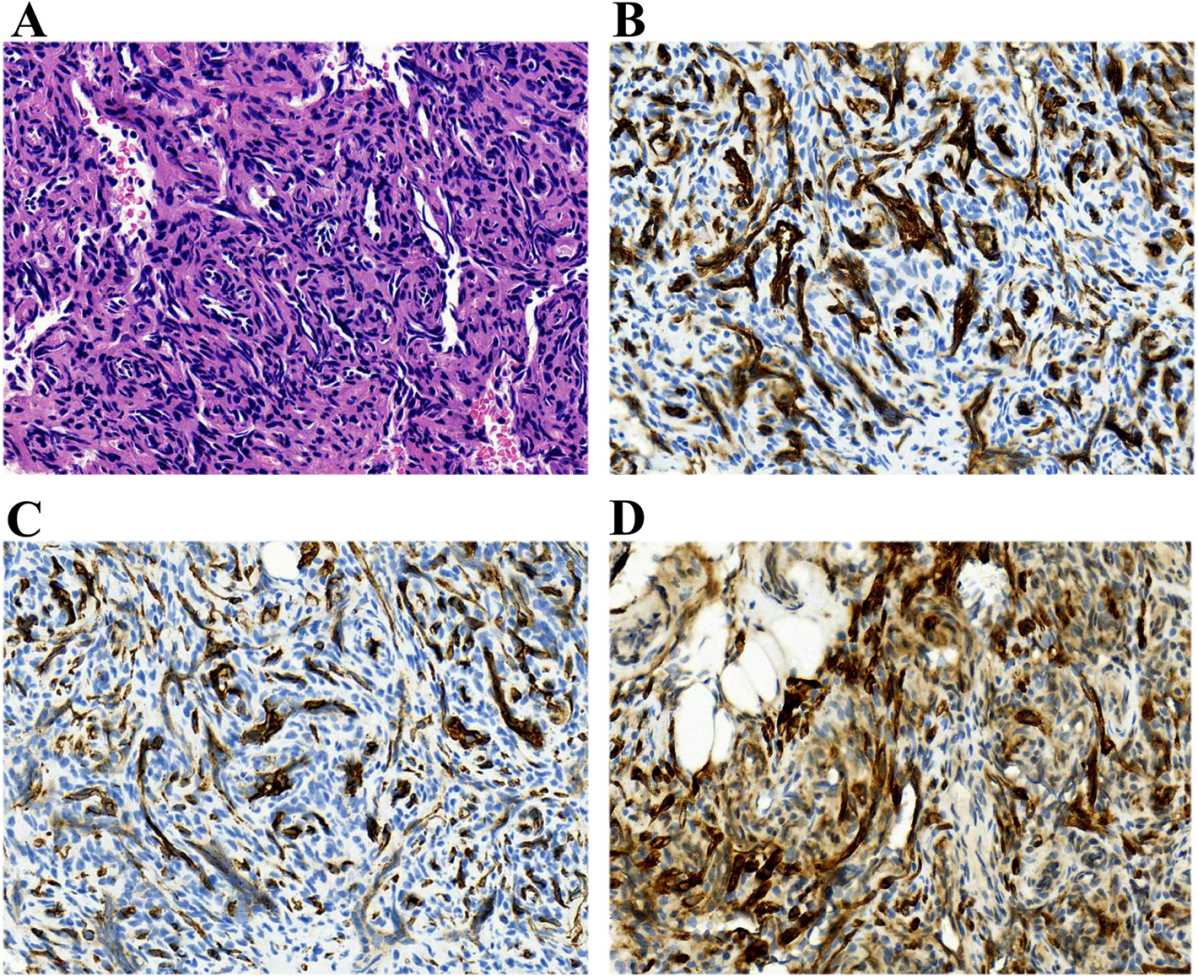
Pathological features of KHE. **a** Haematoxylin and eosin (H&E)-stained showed the lesion was composed of spindle endothelial cells composing abnormal lymphatic channels and slit-like vascular channels. Immunohistochemical staining were positive for vascular endothelial markers CD31 (**b**) and CD34 (**c**), and lymphatic endothelial marker D2-40 (**d**) (original magnification: ×100)

**Fig. 2:**
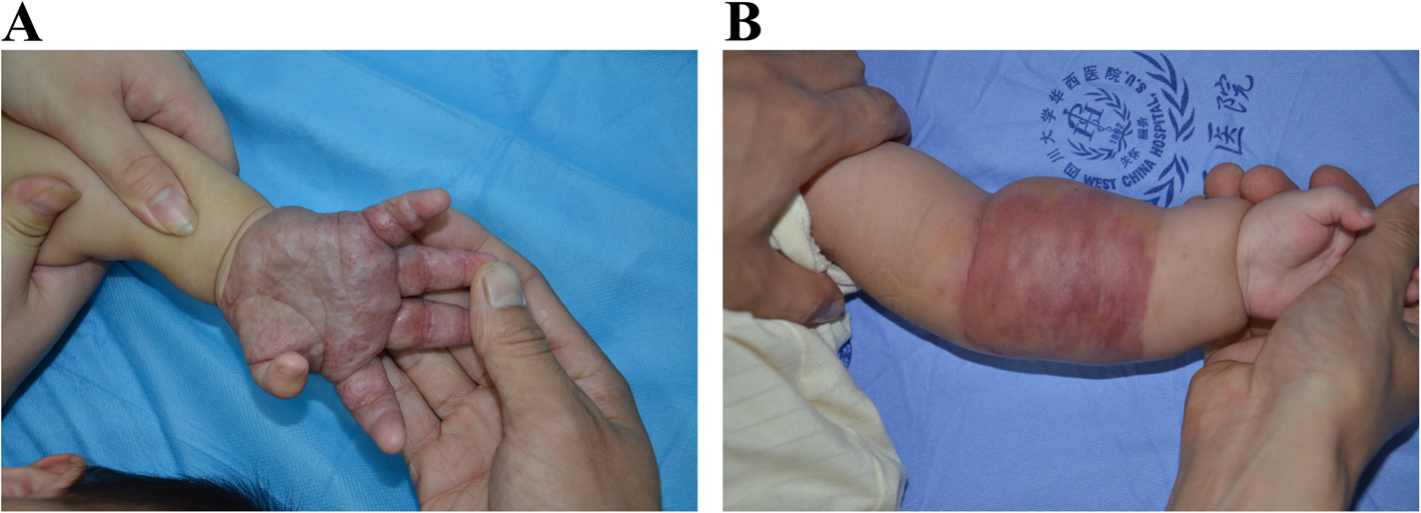
Clinical features of KHE with or without KMP. **a** The patient was found to have a swelling mass on the left hand. The mass became progressively indurated and purpuric. **b** The patient was found to have a vascular lesion on the left forearm. The lesion was bluish in colour and firm

Sixty-four patients were identified through the pathology database as having KHE (Fig. 1). All patients had cross-sectional imaging before tumour resection or biopsy. The cases could be split into two distinct groups: <6 months and ≥ 6 months. The clinical and imaging characteristics of all patients are presented in Table 1. Of the 64 patients with KHE, 36 (24 male/12 female) were < 6 months and 28 patients (18 male and 10 female) were ≥ 6 months. In patients whose age were < 6 months, there were 11 superficial KHEs and 25 mixed KHEs. Physical examination revealed that signs and symptoms related to musculoskeletal complication included decreased range of motion in 17 patients, compression of structures in 4 patients and a characteristic purpuric, cutaneous lesion in 35 patients (Fig. 2). In patients whose age are ≥ 6 months, there were 5 superficial KHEs, 13 mixed KHEs and 10 deep KHEs. Physical examination revealed signs and symptoms related to musculoskeletal complication in 20 patients, the compression of structures in 3 patients and skin lesions in 23 patients. The musculoskeletal complications included: decreased range of motion (18 patients involved), chronic pain (4 patients involved) and physical deformity (8 patients involved).

**Table 1:** The clinical and imaging characteristics of 64 patients with KHE

Parameters	Age at discovery of tumor lesion <6 (m)	Age at discovery of tumor lesion ≥6 (m)	Total	*P*-values
	*n* = 36	*n* = 28	*n* = 64	
**Patients clinical characteristics**				
Sex				
Male	24 (66.7%)	18 (64.2%)	42 (65.6%)	0.842
Female	12 (33.3%)	10 (35.7%)	22 (34.4%)	-
Lesions types				
Superficial	11 (30.6%)	5 (17.9%)	16(25.0%)	0.244
Mixed	25 (69.4%)	13 (46.4%)	38(59.4%)	0.063
Deep	-	10 (35.7%)	10(15.6%)	<0.001
KMP*				
With	23(63.9%)	4 (14.3%)	27(42.2%)	<0.001
Without	13(36.1%)	24(85.7%)	37(57.8%)	-
Musculoskeletal complication	17(47.2 %)	20 (71.4%)	37 (57.8%)	0.052
Chronic pain	-	4(14.3%)	4(6.2%)	0.019
Decreased range of motion	17(47.2%)	18(64.3%)	35(54.7%)	0.174
Physical deformity	-	8(28.6%)	8(12.5%)	0.001
Skin lesion				
With	35(97.2%)	23(82.1%)	58(90.6%)	0.040
Without	1 (2.8%)	5(17.8%)	6(9.4%)	0.040
The compression of structures				
With	4 (11.1%)	3(10.7%)	7(10.9%)	<0.001
Without	32(88.9%)	25(89.2%)	57(89.1%)	-
Age at discovery of tumor lesion (m)				
Mean (range)	1.92 (range1–5)	30.2 (range 6–144 )	14.3 (range 1–144 )	-
**Imaging characteristics**				
Location				
Head–neck area	7(19.4%)	2(7.1%)	9(14.1%)	0.160
Extremities	16(44.4%)	18(64.3%)	34(53.1%)	0.115
Trunk	5(13.9%)	5(17.9%)	10(15.6%)	0.664
Multiple anatomical regions	8(22.2%)	3(10.7%)	11(17.2%)	0.226
Morphological type				
Solitary lesion	14(38.9%)	18(64.3%)	32(50.0%)	0.044
Infiltrative lesion	22(61.1%)	10(35.7%)	32(50.0%)	0.044
Destructive changes or remodeling of adjacent bone	9 (25.0%)	18(64.3%)	27(42.2%)	0.002
Destruction of the adjacent cortex	8 (22.2%)	18(64.3%)	26(40.6%)	0.001
Injury of the epiphyseal region	1(2.8%)	5(17.9%)	6(9.4%)	0.040
Invasion to near joints	4(11.1%)	10 (35.7%)	14(21.9%)	0.018
Maximal tumor dimension (cm)	7.70 (1.91–15.20 )	5.53 (0.86–9.77 )	6.77 (0.86-15.20)	-
Vascularity	6 (16.7%)	3 (10.7%)	9 (14.1%)	0.497
Edema or swelling	12 (33.3%)	16 (57.1%)	28 (43.8%)	0.057

^*^KMP Kasabach-Merritt phenomemon, *m* month

Laboratory investigations were reviewed in all patients. The frequency of KMP (a life-threatening thrombocytopenia and consumptive coagulopathy) was 42 % on the basis of our study of 64 cases of all KHE. In patients whose ages were < 6 months, there was KMP in 23 patients and 4 patients in the ≥ 6 months group. The Pearson chi-squared test showed the frequency of KMP was statistically significant (63.9 % vs. 14.3 %) between < 6 months group and ≥ 6 months group (*P* = 0.000).

On MRI, in patients whose ages were < 6 months, the average maximum diameter of the tumour was 7.70 cm (range 1.91–15.20 cm). Nine patients had destructive changes or remodelling of adjacent bone. Eight patients had a diagnosis of cortex of bone destruction. One patient had injury of the epiphyseal region. MRI showed that 4 patients had invasion to near joints. KHEs were observed at various locations: head-neck area (*n* = 7, 19.4 %), extremities (*n* = 16, 44.4 %), trunk (*n* = 5, 13.8 %), or multiple anatomical regions (*n* = 8, 22.2 %). Fourteen patients (38.8 %) in this group had a solitary lesion and 22 patients (61.1 %) had infiltrative lesion. Six patients in this group had prominent vascularity. Twelve patients (33.2 %) in this group had oedema or swelling on magnetic resonance imaging. In patients whose age were ≥ 6 months, the average maximum diameter of the tumour was 5.53 cm (range 0.86–9.77 cm). On MRI, 18 patients had destructive changes or remodelling of adjacent bone. Eighteen patients had a diagnosis of cortex of bone destruction. Five patients had injury of the epiphyseal region. MRI showed that 10 patients had invasion to near joints. KHEs were observed at various locations: head-neck area (*n* = 2, 7.1 %), extremities (*n* = 18, 64.3 %), trunk (*n* = 5, 17.9 %), or multiple anatomical regions (*n* = 3, 10.7 %). Eighteen patients in this group had a solitary lesion and ten patients had an infiltrative lesion. Three patients in this group had prominent vascularity. Sixteen patients had oedema or swelling on magnetic resonance imaging.

**Fig. 3:**
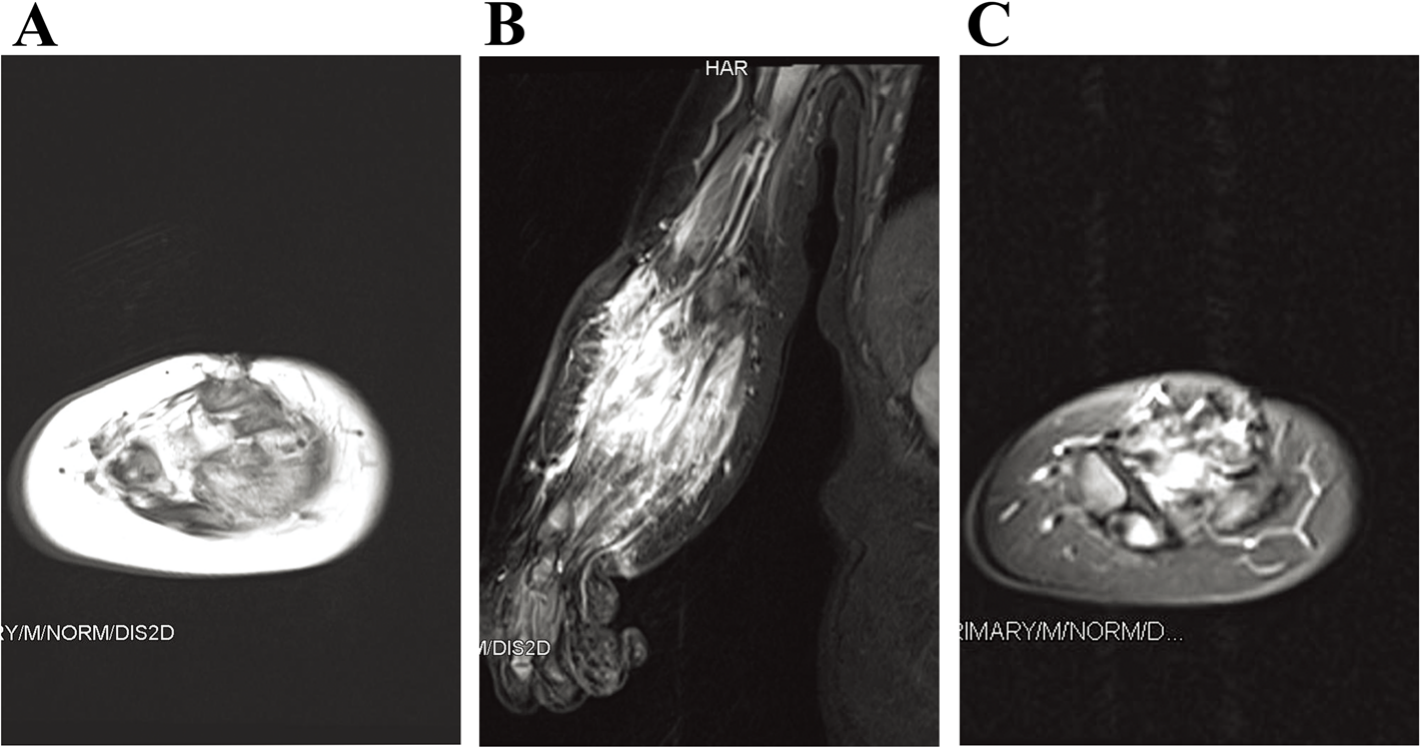
Pathologically confirmed KHE with KMP in a patient. **a**, An axial T1-weighted image reveals an iso-intense signal of the mass to normal muscle. The axial (**b**) and coronal (**c**) T2-weighted images reveal that the tumour has mild hyperintense signal compared to adjacent muscle, the central portion of the mass has bright signal intensity and ill-defined margins, the whole mass shows heterogeneous intense enhancement in the central and peripheral portions. There are engorged vessels in the periphery of the mass and is encircled by a peripheral infiltrative portion that has moderate hyperintensity. The right ulna and radius are involved

**Fig. 4:**
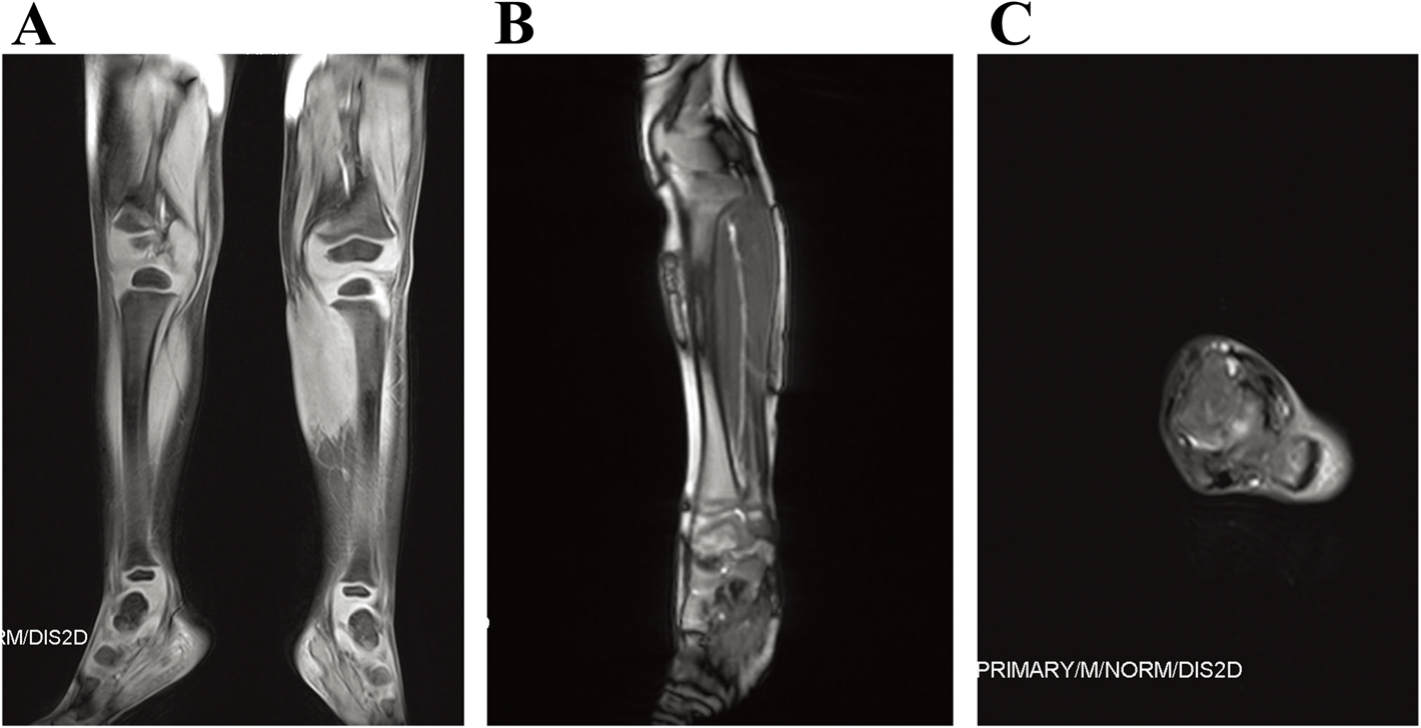
Pathologically confirmed KHE without KMP in a patient. **a** A coronal T2-weighted image reveals that the tumour has hyperintense signal compared to adjacent muscle. **b** An axial T1-weighted image reveals an iso-intense signal of the mass to normal muscle. **c** The horizontal T2-weighted image shows the skin and subcutaneous fat thickened in the middle and upper segment of the left leg

**Fig. 5:**
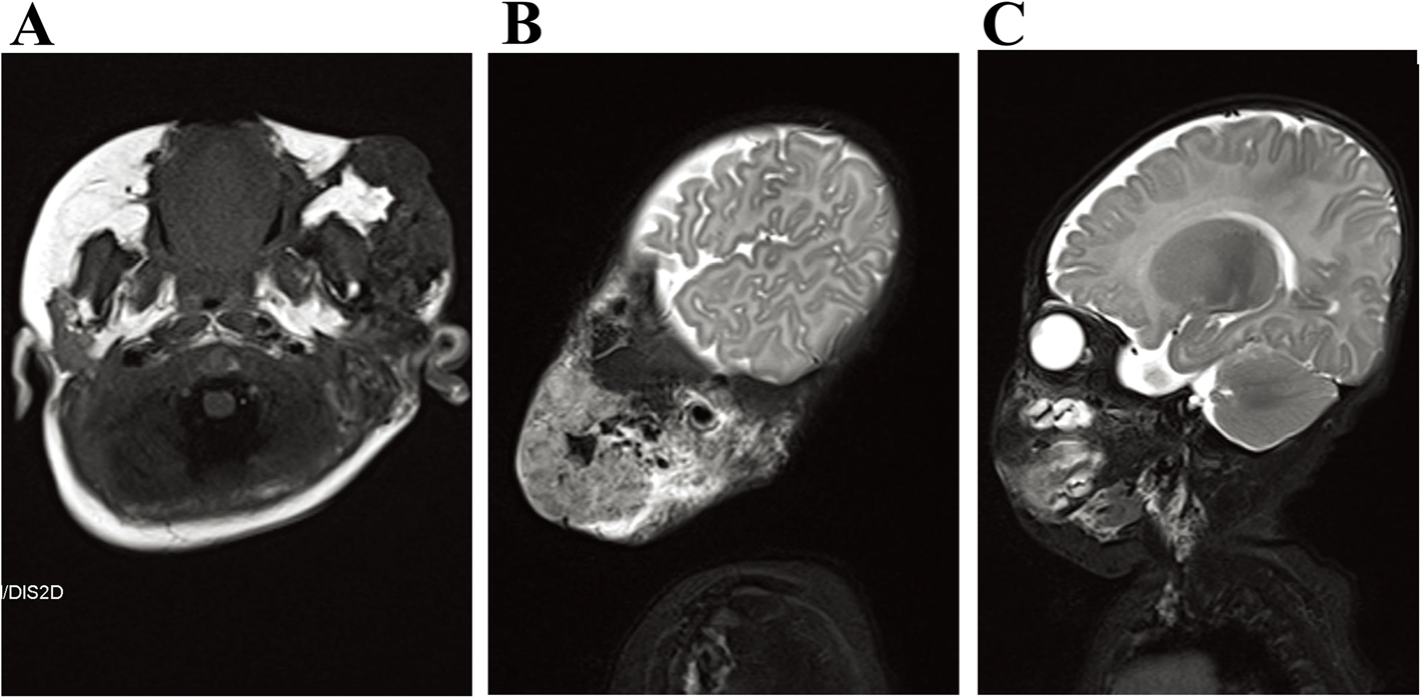
Pathologically confirmed KHE with KMP in a patient. **a** T1-weighted image. **b** and **c** The axial T2-weighted image shows an iso-intense heterogeneous signal of the mass to normal muscle on the left side of the facial skin

**Fig. 6:**
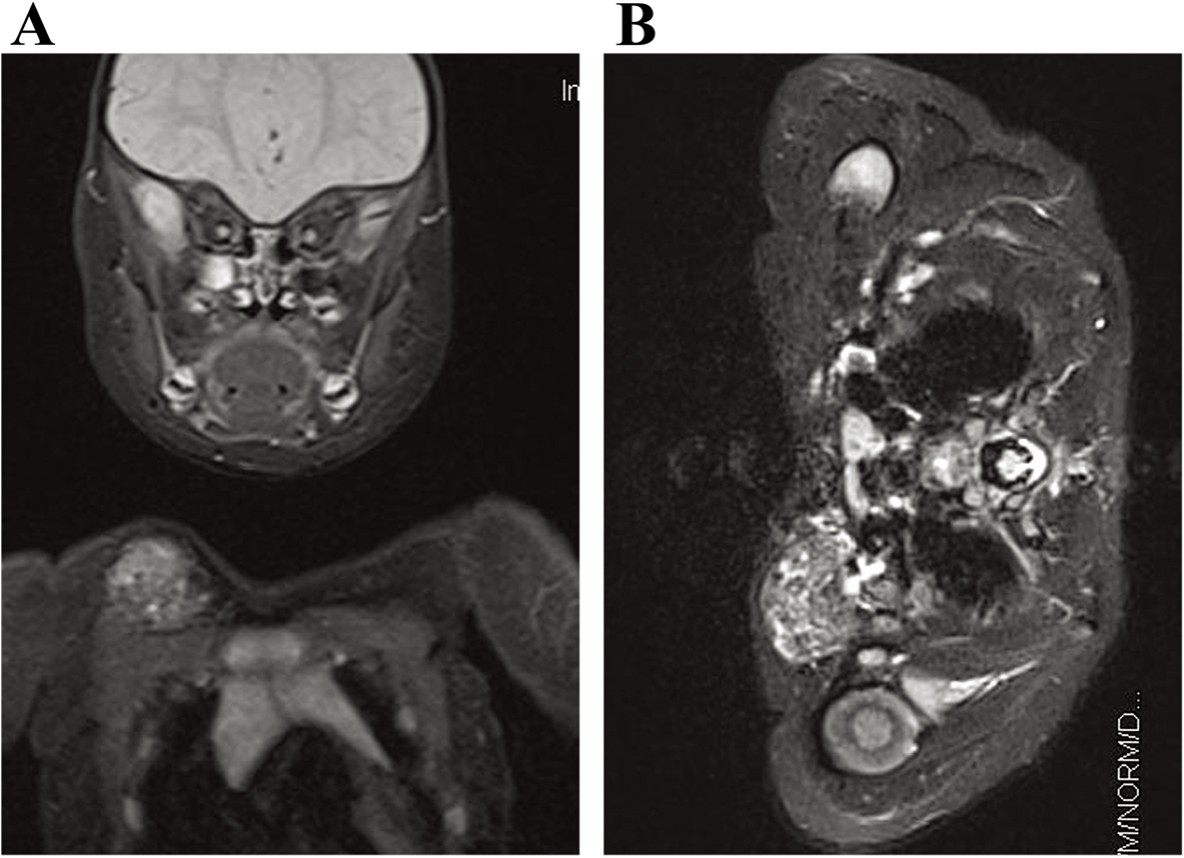
Pathologically confirmed KHE without KMP in a patient. **a** and **b** The T2-weighted image reveals that the mass has with well-defined, heterogeneity, hyperintense signal compared to adjacent muscle at the root of the right neck

The lesions in all of the 64 patients were hypointense or isointense compared with muscle on T1-weighted sequences, and hyperintense on T2-weighted or inversion-recovery sequences (shown in Figs. 3, 4 and 5). Prominent vascular channels (vascularity) were evident as flow voids in the mass or as linear enhancing channels adjacent to the tumour in 9 patients. Cutaneous thickening or enhancing subcutaneous stranding was evident in 16 patients (shown in Fig. 5). Fourteen patients have images consistent with mixed signal within the tumour on T2 signal void (shown in Figs. 3 and 6).

## Discussion

KHE is a rare disease whose prompt and accurate diagnosis may be difficult for clinicians. It has been reported that a KHE diagnosis was delayed by ≥ 1 month in 65.7 % of patients with KMP [2]. KHE shares overlapping patterns of age of presentation and clinical symptoms, anatomical location with other vascular anomalies and tumours such as congenital haemangioma, venous malformation, lymphatic malformation, sarcoma, infantile fibrosarcoma, neuroblastoma and fibromatosis [6, 10]. However, KHE is histologically distinct from other vascular neoplasms [11]. Due to associated complications, KHE has high morbidity rates. In patients with KMP, life-threatening bleeding and compression of vital structures may occur [1, 2]. Furthermore, in patients with KHE without KMP, musculoskeletal complications may lead to disability and influence patients’ quality of life [8, 12]. However, lack of detailed description of imaging findings of KHE in the literature might lead to delayed consideration of the diagnosis. Imaging examination might avoid the risk of bleeding associated with biopsy [13]. In addition, imaging examination could monitor of therapeutic response or follow-up.

The frequency of KMP was 42 % on the basis of our study of 64 cases of all KHE. Consistent with previous study, we indicated that age is a risk factor for the development of KMP. Other factors contributed to the development of KMP include: mixed lesion type and large lesion size [2]. Compared with solitary subtype KHE, the diffusive infiltrative ones were more commonly accompanied by KMP [7]. Based on our findings, we found that those whose lesions were discovered at < 6 months had higher odds of diffusive infiltrative KHE subtype and mixed subtype KHE, compared with the patients whose lesions were discovered at ≥ 6 months. Moreover, the infiltrative imaging characteristics can distinct KHE from other benign vascular tumour, such as infantile haemangioma and congenital haemangioma.

In the present study, we found that the frequency of musculoskeletal complications was 58 %. For patients whose lesions were discovered at ≥ 6 months of age, signs and symptoms related to musculoskeletal complication could become more noticeable. At the same time, it’s noteworthy that patients older than 6 months of age had higher odds of destructive changes or remodelling of adjacent bone. In addition, we revealed that the imaging findings of the adjacent bone changes include destruction of the adjacent cortex, injury of the epiphyseal region and invasion to near joints. The imaging features of KHE were similar to those of kaposiform lymphangiomatosis (KLA) with involvement of multiple planes. Unlike KLA, cutaneous involvement is common in KHE. In our cases, the frequency of the cutaneous involvement was 90.6 %. Furthermore, KLA is almost always multifocal, whereas KHE is most often unifocal [14].

In previous studies, the authors demonstrated that MRI findings of KHE included ill-defined borders, involvement of multiple tissue planes with cutaneous thickening and stranding of the subcutaneous fat, less prominent superficial vessels, and destructive changes of adjacent bone [15]. In our study, most of the maximum diameters of the lesions (42/64) presented more than 5 centimetres. A more explicit description of the adjacent bone changes has been given, including cortex of bone destruction, injury of the epiphyseal region and joints. We also revealed that the diffusive infiltrative KHE subtype in 50 % patients. Based on the imaging findings, the frequency of the mixed subtype KHE was 59 %. As infiltrative KHE subtype and mixed lesion type are important predictors of KMP [1, 2], MRI plays an essential role in discriminating infiltrative KHE subtype and mixed subtype KHE.

Microscopically, KHE is composed of infiltrating nodules with slit-like or crescentic vessels that are poorly canalized and lined by spindled endothelium cells. Dilated hyperplastic lymphatic channels and lymphatic spaces can be seen in KHE lesions, and this has been called ‘‘lymphangiomatosis” [16]. In previous studies, lymphoedema has been reported as a potential sequela of KHE, particularly in a limb location [13, 17, 18]. T2-weighted MRI usually shows a hyperintense reticular network of dilated subcutaneous channels between the dermis and fascial plane  [13, 17]. In patients with KHE, if the primary anomalies of the lymphatics lead to insufficient vessels to drain lymph from the extremity, the lymphoedema may occur [13, 18]. However, the patient’s lymphatic system may develop normally but the tumour mass influences lymphatic development or damages the lymphatic vasculatures. It is hypothesized that the mechanical obstruction of the lymphatic flow during the acute phase of KMP may lead to lymphoedema years later [13, 18]. For patients whose lesions were discovered at ≥ 6 months of age, signs and symptoms related to musculoskeletal complication could become more noticeable. In the present study, swelling/oedema of soft tissue was common. The MRI showed that 43.7 % patients had swelling/oedema of soft tissue. In addition, lymphatic spaces were common in the residua, as was dense fibrosis destroying the reticular dermis and extending deep to fascial layers. In this regard, magnetic resonance lymphangiography has helped us better characterize the lymphoedema anatomy and pathophysiology.

## Conclusions

KHE occurs mostly in infants and in various locations. Presence of an unfamiliar mass exhibiting ill-defined margins, intense heterogeneous enhancement, multi-compartment involvement, adjacent fat stranding, destructive changes of adjacent bone, swelling/oedema of soft tissue, with or without KMP should favour the diagnosis of KHE.

## Data Availability

The data used during the current study are available from the corresponding author on reasonable request.

## Abbreviations

KHE: Kaposiform haemangioendothelioma
KMP: Kasabach-Merritt phenomenon
US: Ultrasound
MRI: Magnetic resonance imaging
KLA: Kaposiform lymphangiomatosis
